# How Cool Is That: An Interview with Caroline Dean

**DOI:** 10.1371/journal.pgen.1003593

**Published:** 2013-06-27

**Authors:** Jane Gitschier

**Affiliations:** Departments of Medicine and Pediatrics and Institute for Human Genetics, University of California San Francisco, San Francisco, California, United States of America

Having grown up in Pennsylvania, I recall that late winter's delight in spotting a purple crocus piercing through desiccated gray snow, a harbinger for warmer days ahead. The eruption of the crocus, the bloom of the magnolia, and the flowering of winter wheat are all examples of plant processes that are not simply delayed by winter, but indeed *require* a sustained period of cold to proceed. Today's interview journeys into the molecular underpinnings of one such cold-dependent process: “vernalization.” Our guide is Caroline Dean from the John Innes Centre in Norwich in the United Kingdom, and our destination is an ∼10-kb genomic stretch of the humble *Arabidopsis thaliana*, a small white-flowering bit of greenery also known variously as thale or mouse-ear cress.

A little background is in order here, as perhaps you, like me, had never heard the term vernalization. During the development of flowering plants, shoot growth occurs at the apical meristem, an undifferentiated mass of cells that can divide to make more stems, leaves, or flowers. While some plants are “rapid cyclers”—i.e., they flower quickly after a short growth period—others require a duration of cold before they can flower, an evolutionary adaptation that allows them to resist flowering until the winter has ended. Often, varieties within a single species can exhibit one behavior or the other, such as spring and winter wheat, only the latter of which requires vernalization. Accessions of *Arabidopsis* vary in their vernalization requirement too, depending upon whether they grow along the Mediterranean coast or above the Arctic Circle, for example.

A few decades ago, when *Arabidopsis* emerged as the plant of choice for molecular geneticists, Dean (Image 1) began to question how vernalization works at the mechanistic level: what genes are required for vernalization and how do vernalizing plants sense and remember the cold? She and her colleagues attacked the problem via three genetic routes, all of which ultimately converged on the regulation of a single gene, *FLC (FLOWERING LOCUS C)*, whose gene product is a repressor of downstream flowering genes. The “overwintering” accessions respond to cold by epigenetically silencing *FLC*, enabling flowering to occur. This silencing involves a conserved Polycomb complex and associated PHD (plant homeodomain) proteins that modify histones at the *FLC* locus. The *FLC* locus also produces antisense transcripts known as “*COOLAIR*,” which differ in their splicing and polyadenylation sites, and these assist in locking down *FLC* transcription in the cold. Once *FLC* is silenced, flowering can begin in earnest with the advent of longer and warmer days. These epigenetic changes are maintained throughout future cell divisions and even persist in cuttings regenerated from the vernalized plants so that they do not need to be re-vernalized. However, when a seed is being produced, *FLC* silencing is removed so that the next generation of plants also requires vernalization.

**Figure:**
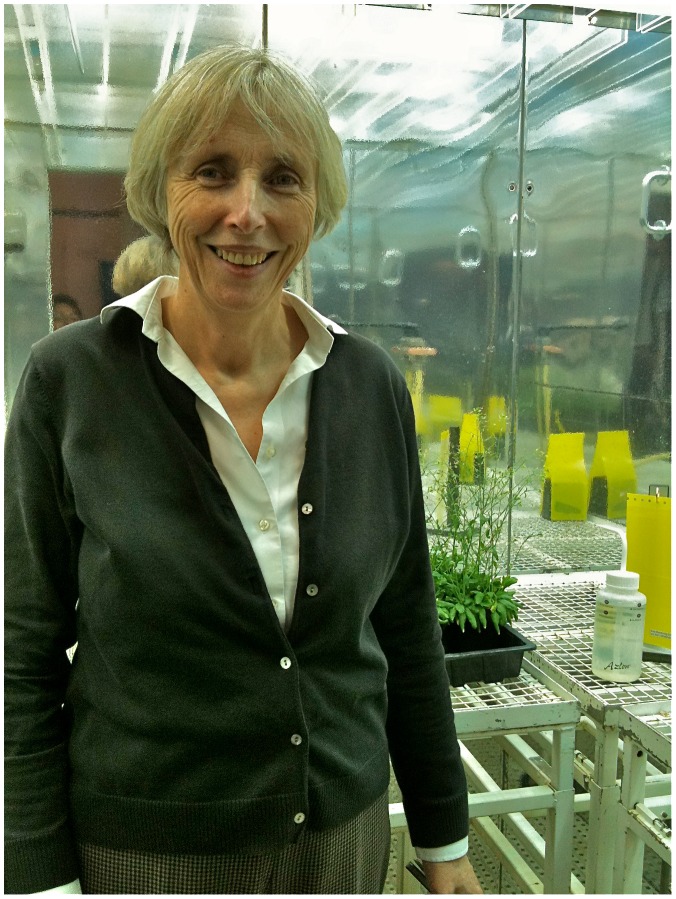
Caroline Dean. Photograph by Jane Gitschier.

I learned about Dean's work just as I was heading to the UK and quickly arranged to visit her on a Wednesday afternoon in November. It was certainly my most active interview to date, as we scooted out to the “barns” to see *Arabidopsis* and its *Brassicaceae* cousins—broccoli, rapeseed, cauliflower—in all manner of leafing and flowering. This involved some robing and disrobing of bright red lab coats and hairnets with much humor along the way. It has taken me a little while to digest this complex subject, but following its own “overwinter,” the interview has now gone on to flower.

**Gitschier:** Let's start with your choice of a postdoc and how you got involved in the question of vernalization.

**Dean:** I started off doing plant biology at York University. I did my PhD work on chloroplasts and then I wondered what on earth I would do as a postdoc. My supervisor had been on a sabbatical to Riverside [at the University of California], and she *raved* about it. I remember thinking gosh, it sounds really exciting over there.

It was just at the time—1982—when it was discovered that a piece of the DNA from the plasmid in *Agrobacterium* [*tumefaciens*] could be inserted into the plant chromosome. So, genetic engineering was born. A lot of money went in from venture capital to start up new biotech companies, and John Bedbrook from New Zealand—the person who had cloned the first plant gene—was persuaded to start one of the new companies in Berkeley: Advanced Genetic Sciences. AGS is famous for putting engineered bacteria on strawberries as an ice-protection measure called “Frostban.” It created a big stir in California because it was the first release of a genetically engineered organism. John offered me a position and I grabbed it.

**Gitschier:** Very exciting time.

**Dean:** You know, it was a dream. I arrived in February—[in England] it was cold, very dark, I had hardly any money. Then suddenly, here I was in California. They loaned me a car. The salary seemed enormous. And sunshine!

**Gitschier:** Actually, our paths are quite similar because I also did a postdoc in California with a young company at about the same time. It was the best four years of my life, without a doubt.

**Dean:** Yes. Coming from a small plant lab in York, suddenly being exposed to this amazing amount of information and technology and “can-do” attitude, I was just transformed *bang!* into a new way of doing science.

We had five successful years establishing the fundamentals behind genetic engineering. And then, slowly but surely, financial reality set in for these biotech companies. The venture capital money ran out, and research contracts with very specific goals replaced it. At that point it was a “we” rather than a “me,” so we looked around for academic positions, and we took two here in Norwich. It was home, but also the Sainsbury Lab had just been set up. Jonathan [Jones, Dean's husband] got the position there, and I came back to the position at the John Innes.

**Gitschier:** For me, going back to academia from industry was appealing because it was a new kind of a challenge. In industry, I felt I knew what was expected of me, whereas in academia, it was a question of how creative I might be.

**Dean:** Yes, that's right. I started to plan what to do in my own lab, and the answer came from a sort of bizarre experience in California. I wanted to have a few bulbs in my apartment. So I bought some tulips, and the sales chap said to me, “Now don't forget to put them in the fridge for six weeks.” Of course, in England, you sow them in the ground in September, October, but you never think “why.” This was how I learned about the requirement for prolonged cold for many plant processes: flower emergence in plants like tulips, breaking bud dormancy in trees, and vernalization in many plant species.

Now vernalization is not quite the same process as the need for cold in tulips. In the [tulip] bulb, the flower has already initiated before the cold, but its growth is arrested; the low temperature is important for stalk elongation. A similar break in late developmental arrest by prolonged cold also occurs in the case of spring blossoms in trees. In each case you need a couple of months of cold. It is an evolutionary adaptation to make sure you don't fully flower until spring. We still don't know really much about all of those processes except for vernalization.

Vernalization is very important for many crops, and winter and spring varieties have been bred independently. All the cereals—barley, wheat, rye—have winter and spring varieties that the farmer sows either in October or in March. And a lot of the *Brassicas*, of course, most of the vegetables, actually have a vernalization requirement. And what breeders have done is to breed for the need for different lengths of cold, so that you then have a nice supply for the supermarket year-round.

**Gitschier:** Well, the old-fashioned cauliflower, before anybody bred in anything, when would that have appeared? The spring?

**Dean:** Have you ever left a cauliflower or broccoli too long and then it's sprouted? You actually get a beautiful flower coming out of the top.

**Gitschier:** No, but I'm keen to do that experiment!

**Dean:** So, it's an arrested meristem, the bit we eat.

**Gitschier:** The bit we eat is an arrested meristem!

**Dean:** Cauliflower is an earlier arrest than broccoli, sort of little stubs, and if you leave them longer, each one of the many buds turns into a proper flower.

**Gitschier:** Cool.

**Dean:** For the last couple of years, we've had very extreme winters, but not always at the same time. Last year after Christmas we had very, very cold temperatures. The year before, there was a famous satellite picture of the UK *all* under snow before Christmas. Too much cold at any point can mess up the scheduling of the broccolis and the cauliflowers because they vernalize way too quickly and come into heading at about the same time. So suddenly there was a glut of cauliflower and then months when there was no production in the UK after that.

**Gitschier:** Ah, so it's not just the duration of the cold but also the actual temperature. This is very complex.

**Dean:** It is. The current thinking describes vernalization efficiency in terms of the “day degree” exposure, but this is poorly understood. We also don't know the temperature optima for the different varieties—they are probably different.

**Gitschier:** Well that triggers a pressing question, but before I ask it, let's discuss the molecular definition of vernalization.

**Dean:** For *Arabidopsis*, vernalization is the process by which *FLC*—a floral repressor gene—becomes epigenetically silenced. *FLC* is one of many factors that regulate when a plant flowers.

A good analogy is to think of *FLC* as a car brake. You're in a car, you have your foot hard on the brake, you won't move—you won't flower—even if you are pressing the accelerator with long day photoperiods and warmth, you're not going to flower.

But over winter, slowly but surely, you take your foot off the brake. It takes a long time. When you come into spring, if you haven't got warm days, you don't actually flower, but you have the *ability* to flower because you are now *not* having a foot on the brake. Now you wait for the photoperiod to get longer, you have temperatures increase, now you are putting your foot on the accelerator.

Vernalization, then, is to epigenetically silence *FLC*.

**Gitschier:** Equivalent to taking your foot off the brake to flowering.

**Dean:** Yes. And the interesting thing is that it is a quantitative effect. How can one week of cold not be as good as two weeks or three weeks? That has been a mystery—how can you *quantitatively* silence something? It's because of progressive switching of bistable epigenetic states, from one that promotes expression to another associated with silencing after cold exposure.

We showed that the cold causes a slow accumulation of PHD proteins at the *FLC* locus. These proteins associate at one site in the *FLC* locus with the Polycomb repressor complex 2 [PRC2], which is already poised at the locus. We think the PHD proteins somehow supercharge the activity of PRC2, just as Juerg Muller, working on an analogous complex in Drosophila, finds increased activity *in vitro* of the methyltransferase if a PHD protein associates with PRC2. And the longer the plants stay in the cold, we see more methylation of lysine 27 on histone 3 [H3K27me3, the classic histone modification associated with Polycomb silencing] at that site. This probably explains why vernalization is so slow and why six or eight weeks of cold gives more H3K27me3 at that site compared to say two weeks.

Once plants begin to feel warm again, the PHD proteins zipper along the whole length of the gene, increasing H3K27me3 along the whole gene. This is needed for mitotic stability of the silencing through the rest of development.

**Gitschier:** So, for the tulip bulb, say, you're slowing down not necessarily this *FLC* switch, but something else.

**Dean:** Yes, it would be a different regulator, but probably via a similar mechanism.

**Gitschier:** Are people working on that? Are there tulip molecular biologists?

**Dean:** There are people looking at a similar question in trees. Many buds in trees are arrested in autumn, and they have gone in and said, “If I knock out the Polycomb, do I change bud dormancy?” And yes they do. So it is clearly following the Polycomb paradigm. They don't know the target, but they know the mechanism.

OK, it's getting dark, time to go to the barn!

[My pressing question will have to wait! We take a brisk walk to the barn. Upon arriving things get animated.]

**Dean:** We have to put on…

**Gitschier:** Oh, we have to suit up to see the plants!

**Dean:** We do! This is for your head [as Dean hands me a hairnet]. We had a terrible time with a virus which is carried by a little insect called a thrip, which of course gets in your hair.

**Gitschier:** Oh God, I don't want that.

**Dean:** Well, we're preventing the thrip getting *in*! We're protecting the plants!

[Next we meet George.]

This is Jane from San Francisco and we've come to see the plants. This is George Lomonossoff.

**Gitschier:** Now George, once we get suited up, will you take a picture of the two of us together?

**Dean:** You have to promise me—there is *no way* we can have this published!

**Gitschier:** I promise you. [This is so much fun!]

**George:** OK, smile.

[Click. We enter the barn.]

**Gitschier:** Oh my God this is beautiful! I want to work here! Now, does the barn house your research only?

**Dean:** No, as we go round we'll see lots of other things. We have petunias because they are very, very sensitive to the virus. They will come down before the *Arabidopsis*. They are sentinel plants.

**Gitschier:** Oh, I love that! OK, let me just take a picture of a sentinel petunia. [Click.]

**Dean:** These are *Arabidopsis*. There are winter and spring types, you see. Some of them are very late flowering, some are early flowering. Look at these for late flowering: they are so late! They keep making more leaves! Instead of just making five or six leaves and then flowering, they make over a hundred leaves before flowering. They are so squished.

**Gitschier:** Now what is the gene that is wrong with them?

**Dean:** *FLC*! These are transgenic plants where we added the *FLC* allele from plants from Northern Sweden. Without cold, it is *such* a strong brake; they just stay vegetative without cold until we vernalize them.

**Gitschier:** This is great. And “we vernalize them” how?

**Dean:** I'll show you the vernalization room.

**Gitschier:** Now what is going on with these? They look like vines.

**Dean:** When *Arabidopsis* flowers, it produces an inflorescence from which the flowers emerge. They self-fertilize and seed is produced. So what someone has done here is stake them up because they are trying to collect as much seed as possible.

**Gitschier:** How did you get interested in *Arabidopsis* [as we meander]?

**Dean:** I heard Chris Somerville's postdoc, Jose Martinez-Zapater, give a talk in 1987 about late-flowering mutants in *Arabidopsis*. But actually, if you vernalize these late-flowering mutants, they flower early again! As soon as I heard this, I thought, “This is how I can dissect vernalization. Here is genetic variation that will be my entry point.” That recessive vernalization requirement turns out to identify the “autonomous” pathway: Loss-of-function of a whole set of genes—*FCA*, *FPA*—causes late-flowering through de-repression of *FLC*. This pathway involves alternative processing of the *FLC* antisense transcripts.

This mechanism contrasts with the dominant vernalization requirement that we identified through analysis of natural variation in flowering time in *Arabidopsis*. This involves a protein called FRIGIDA that upregulates *FLC*. The rapid-cycling *Arabidopsis* types have mainly arisen through independent loss-of-function mutations at *FRIGIDA*.

And then our third tack was to mutagenize vernalization-dependent *Arabidopsis* and look for mutants that no longer vernalize. These were the *VRN* mutants, and that's what got us into the Polycomb world.

**Gitschier:** It's amazing, actually, that it all points to this one gene, *FLC*.

**Dean:** It is! Slight changes in *FLC* expression affect the flowering time, and any change in flowering time affects seed yield or fitness, so the trait will be under enormous selection.

[We leave the *Arabidopsis* part of the barn, crashing into a science review panel who are having entirely too much fun.]

**Gitschier:** There is a party out here!

**Dean:** This is Jane from San Francisco, from *PLOS Genetics*. This is half the science review panel from BBSRC!

**One of the panel:** And you're lovely! [It's the hairnets, I'm sure. We move on to cauliflowers and strip away our barn accoutrements.]

**Gitschier:** We don't need red coats for the cauliflowers?

**Dean:** No, just for the *Arabidopsis*. Here we've got rapeseed, *Brassica napus*, which gives you those wonderful yellow fields. It is a very close relative of *Arabidopsis*.

**Gitschier:** What about these guys?

**Dean:** These are *Antirrhinum*—snapdragon.

And here is broccoli. The breeders have made hybrids and selected ones with varying requirements for cold. We are now figuring out that certain combinations of genes have been selected. By knowing all this, we can develop varieties with particular cold requirements. And the same for cauliflower.

And *this* is the vernalization room.

[Ah! The mecca. Like any cold room, but *ver*y cold and *very* dark.]

**Gitschier:** Yikes! How do people work in here?

**Dean:** Well, they don't really. The lights are on only until 4 o'clock.

**Gitschier:** How do these things even exist over the winter anyway?

**Dean:** They are amazingly healthy under the snow for months. I am fortunate to be part of a big field experiment in Sweden, and each year, I wonder whether these plants are going to survive under snow for four or five months. But they do.

**Gitschier:** Now, that is one cold room! [As we exit and head back to the lab.]

**Dean:** You don't want to be in there for very long.

**Gitschier:** And now for my pressing question! What *is* it about the cold? How does the cell *recognize* cold?

**Dean:** Well, that's still one of the big questions. There are many different, independent temperature steps involved in *FLC* silencing and we'd very much like to figure it all out at the molecular level. If I had to guess, it could be secondary structure of RNA, which is involved in the core chromatin mechanism. There are antisense RNAs from the *FLC* locus, and I think in the end, when we really get down to the “what is it that perceives the temperature,” it may well be an RNA thermosensor.

**Gitschier:** That would be something! RNAs altering their secondary structure in response to temperature and these changes having some mechanistic effect on the locus.

**Dean:** There are clearly several different temperature steps that directly target *FLC*. So another question is how do these different thermosensors integrate different aspects of that temperature?

**Gitschier:** How did you discover these antisense transcripts?

**Dean:** We wanted to identify an early cold-induced step that occurs before the induction of PHD proteins. Given the excitement of small RNAs in chromatin regulation, we asked whether there is a role for noncoding RNA in vernalization too. So we made a custom array of *FLC*, a high density, single-nucleotide resolution custom tiling array that included *FLC* and neighboring genes, and probed that with different RNAs extracted from plants at different stages of vernalization and of different genotypes.

**Gitschier:** And you tiled both strands?

**Dean:** Yes, because we didn't really know what to expect.

**Gitschier:** But how did you know there was even going to be a noncoding RNA in there?

**Dean:** We didn't! But it just jumps straight out. There is a massive upregulation in antisense transcripts in plants with two weeks' cold. [She shows me the data.]

**Gitschier:** Wow!

**Dean:** And we called them “*COOLAIR*” transcripts, based on the “HOTAIR” transcripts [“homeobox transcript from intergenic regions”], which were named in Howard Chang's lab at Stanford and are involved in mammalian Polycomb silencing. We thought, “How funny!”

It turns out the *COOLAIR* transcripts are also important in regulating *FLC* in the warm. And the 3′ processing factors that function in the autonomous pathway, independently of vernalization, target *COOLAIR* too. That was a total eureka day.

**Gitschier:** When was that eureka day?

**Dean:** About three years ago.

**Gitschier:** It is really heating up!

**Dean:** It *is* really heating up! And suddenly noncoding RNA is everywhere. I think that's why *FLC* regulation is intriguing to other scientists, because it's providing some important concepts for conserved mechanisms. We have lots of genetic tools and also have the natural variation that allows for molecular and biochemical dissection.

**Gitschier:** So, this temperature thermometer then involves specific sequences in these noncoding RNAs?

**Dean:** Well, that's my favorite hypothesis. We are exploiting natural accessions which are adapted to northern Sweden and need twelve weeks cold compared to only four weeks needed by accessions further to the south. Big, big difference! We've done the genetics and mapped the determinants of the time difference down to the *FLC* locus. In fact, it's down to four noncoding polymorphisms close to the region that this PHD-PRC2 binds to in the cold. We're now looking into whether these polymorphisms affect *COOLAIR* secondary structure. Could that then somehow influence the association of these different Polycomb complexes?

We have a lot of experiments going on in Sweden, where we've asked: if you plant in the wrong place, can you really show that all of the variation, say from those four polymorphisms, is adaptively important? And I think the answer will be “Yes.” We can get at the really detailed mechanism that is underlying the little changes that have allowed these things to adapt to very different places.

To go out and actually see it in the field, to understand the arrangement of the alleles with the environmental conditions and their fitness consequences: that's what I love.

